# Shuangshen Ningxin capsule alleviates myocardial ischemia–reperfusion injury in miniature pigs by modulating mitophagy: network pharmacology and experiments in vivo

**DOI:** 10.1186/s13020-023-00810-z

**Published:** 2023-09-20

**Authors:** Feifan Jia, Yuanyuan Chen, Gaojie Xin, Lingmei Li, Zixin Liu, Sujuan Xu, Jiaming Gao, Hongxu Meng, Yue Shi, Yanlei Ma, Lei Li, Jianhua Fu

**Affiliations:** National Clinical Research Center for Chinese Medicine Cardiology, Xiyuan Hospital, Chinese Academy of Chinese Medical Sciences, Beijing, China

**Keywords:** Myocardial ischemia–reperfusion injury, Shuangshen Ningxin capsule, Network pharmacology, Molecular docking, Mitophagy

## Abstract

**Background:**

Myocardial ischemia/reperfusion injury (MI/RI) is involved in a variety of pathological states for which there is no effective treatment exists. Shuangshen Ningxin (SSNX) capsule which is developed by Xiyuan Hospital, Chinese Academy of Traditional Chinese Medicine has been demonstrated to alleviate MI/RI, but its mechanism remains to be further elucidated.

**Methods:**

The MI/RI miniature pigs model was constructed to assess the pharmacodynamics of SSNX by blocking the proximal blood flow of the left anterior descending branch of the cardiac coronary artery through an interventional balloon. The principal chemical compounds and potential targets of SSNX were screened by HPLC–MS and SwissTargetPrediction. The targets of MI/RI were identified based on Online Mendelian Inheritance in Man (OMIM) and GeneCards. Cytoscape 3.9.0 was applied to construct a protein–protein interaction (PPI) network, and Gene Ontology (GO) functional annotation and Kyoto Encyclopedia of Genes and Genomes (KEGG) pathway enrichment analysis were performed using metascape. To further validate the mechanism of SSNX, Molecular docking, Transmission electron microscopy, and Western blot analysis were used to test the effectiveness of targets in related pathways.

**Results:**

Our results indicated that SSNX significantly improved cardiac function, attenuated myocardial I/R injury. Through network analysis, a total of 15 active components and 201 targets were obtained from SSNX, 75 of which are potential targets for the treatment of MI/RI. KEGG and MCODE analysis showed that SSNX is involved in the mitophagy signaling pathway, and ginsenoside Rg1, ginsenoside Rb1 and ginsenoside Rb2 are key components associated with the mitophagy. Further experimental results proved that SSNX protected mitochondrial structure and function, and significantly reduced the expression of mitophagy-related proteins PTEN-induced putative kinase 1 (PINK1), Parkin, FUN14 domain containing 1 (FUNDC1) and Bcl-2/E1B-19 kDa interacting protein 3 (BNIP3) in MI/RI miniature pigs.

**Conclusion:**

In our study, the integration of network pharmacology and experiments in vivo demonstrated that SSNX interfered with MI/RI by inhibiting mitophagy.

**Supplementary Information:**

The online version contains supplementary material available at 10.1186/s13020-023-00810-z.

## Introduction

Acute myocardial infarction, a critical condition of coronary atherosclerotic heart disease, poses a major risk to human health [1]. Restoring perfusion is the most effective method for treating acute myocardial infarction; nevertheless, restoring perfusion is a “double-edged sword.” [2]. It will inevitably result in further deterioration of myocardial ultrastructure and cardiac function damage, or even irreversible damage, which is known as myocardial ischaemia–reperfusion injury(MI/RI) [3, 4]. Although some progress has been made in MI/RI, the mechanism are still not fully clarified. As a result, it is essential to investigate safer and more effective therapeutic interventions to reduce the clinical risk of MI/RI and provide new treatment strategies.

Shuangshen Ningxin Capsule(SSNX) is a traditional Chinese medicine composed of ginseng, Salvia miltiorrhiza, and Rhizoma corydalis, the primary active ingredients are ginseng saponins (from Panax ginseng C. A. Mey.), total phenolic acid(from Salvia miltiorrhiza Bge.), and total alkaloids(from Corydalis yanhusuo W.T.Wang) in the ratio of 1:1:1.25. Our laboratory has conducted a lot of research on SSNX, the results showed that SSNX significantly reduced serum cardiac enzyme activity, including serum creatine kinase(CK), creatine kinase-MB(CK-MB) and lactate dehydrogenase(LDH), reduced myocardial infarct size, improved myocardial edema and inflammatory infiltration, and protected rats from myocardial ischemia–reperfusion injury by preserving adenosine triphosphate(ATP), maintaining myocardial cell energy charge levels, and opening mitochondrial ATP-sensitive potassium channels [5, 6]. In addition, SSNX significantly increased cardiac function, improved cardiac hemodynamic parameters, reduced myocardial infarct size, and decreased myocardial injury markers such as CK, LDH, CK-MB and serum cardiac troponin(cTnT) in rats with coronary microcirculation disorders caused by microsphere embolism, thereby improving myocardial tissue injury in rats with coronary microcirculation disorders [7, 8]. However, the main bioactive components of SSNX for MI/RI and their specific protective mechanisms are not clear.

Network pharmacology is systems biology-based discipline that can explain the mechanism of drug-disease interaction and explore the material basis of drug efficacy from a holistic perspective by constructing a “component-target-pathway-disease” network, which is applicable to the characteristics of multi-target and multi-component therapy of Chinese medicine [9] Previous studies have demonstrated that SSNX is effective in ameliorating MI/RI in rats, considering the significant physiological and anatomical differences between rodents and humans that limit its clinical translational potential. In contrast, the physiological structure of the heart in miniature pigs and humans has more similarities than that of rodents [10]. Therefore, in this study, the pharmacodynamic effects of SSNX were first demonstrated by constructing MI/RI mini-pig model, and then a network pharmacology approach was used to explore the potential mechanism of SSNX against MI/RI, which was further explored by molecular docking and relevant target validation.

## Materials and methods

### Drugs and reagents

Shuangshen Ningxin Capsules (20201216) were provided by the Pharmacy Department, Institute of Basic Medical Science, Xiyuan Hospital, Chinese Academy of Traditional Chinese Medicine (Beijing, China); Nicorandil Tablets (17F020Z) were obtained from Chugai Pharmaceutical Company (Beijing, China); Sodium pentobarbital (020402) was provided by Beijing Chemical Reagent Company (Beijing, China); Heparin Sodium Injection (151703010A) was purchased from Qianhong Biochemical Pharmaceutical Company (Changzhou, China); CK assay kit (20210129), ATP assay kit (20201207) and membrane potential assay kit (20201125) were purchased from Jiancheng Institute of Biological Engineering (Nanjing, China); cTnT assay kit (20211205) was purchased from Shanghai Enzyme Link Ltd. (Shanghai, China); LDH assay kit (005113) was purchased from Wako Pure Pharmaceutical Co. Ltd. (Beijing, China); Masson trichrome staining solution (03170101) was purchased from Guangzhou Vigers Biotechnology Co. Ltd. (Guangzhou, China); Hematoxylin eosin(HE) staining solution (1116A18) was purchased from Xinfan Biotechnology Company (Nanjing, China); Nitroblue tetrazolium chloride (20201207) was purchased from Solepipe Technology Company (Beijing, China); The following antibodies were used: PINK1 (23,274-1-AP) and Parkin (14,060-1-AP) were from Benova Biotechnology Co., Ltd. (Beijing, China); GAPDH (Ab109414), FUNDC1 (Ab74834) and BNIP3 (Ab109362) were from abcam Ltd. (Shanghai, China).

### SSNX composition analysis

HPLC experimental conditions: the atomized gas and dry gas are nitrogen, and the collision gas is helium. The capillary voltage is 3500 eV, the atomization temperature is 350 °C, the dry gas is 10.0 L / min, and the atomized gas is 206.85 kPa. The MS scanning range is m/z 80 ~ 1000, and the data storage mode is the centroid. The ion source was double ESI spray, and the data were collected by mass spectrometry through 2 known standards [Hexakis (1H, 1H, 3H-tetrafluoropropoxy) phosphazine And 7H-purine, corresponding to m/z 922.00980 and 121.0509] for real-time correction. The reference solution was injected into the mass spectrum at a rate of 0.01 ml/min by an Agilentisonic pump. The AutoMS/MS experiment uses CID collision. Liquid chromatography is an Agilent 1290 series UHPLC system. The liquid phase is separated by a reversed-phase column ( Atlantis, T3, 150 m m × 2.1 mm, 3 μm,Waters,IRELAND), Mobi le phase A phase is 0.1% formic acid–water, and phase B is 0.1 percent formic acid- (methanol: acetonitrile = 1: 1), Gradient elution conditions under positive ion conditions: 0–40 min, 92% B; 17–32 min, flow rate 0.3 ml/min, column temperature 35 °C, injection volume 10 μl; Negative ion gradient elution conditions: 0–5 min, 33–35% B; 5–40 min, 35% -65% B, The column temperature was 35 °C and the injection volume was 10 μL.

### Pharmacodynamic study of SSNX on MI/RI miniature pigs

#### Animal

5–8 months old male miniature pigs (25–35 kg) were provided by Beijing Shichuang Century miniature pigs Breeding Base, license: SYXK (Beijing) 2018–0004. Animal experimental operations and feeding were conducted at the Experimental Animal Centre of Xiyuan Hospital, Chinese Academy of Traditional Chinese Medicine, License: SYXK (Beijing) 2015–0011.

#### MI/RI miniature pigs model construction and treatment

Our previous study showed that the protective effect of SSNX 90 mg/kg was more significant in MI/RI rats [5, 6], and according to the dose conversion table for standard weight animals [11], the dose for miniature pigs was 20 mg/kg. Based on the above results, 20 mg/kg was selected for the experiment in miniature pigs in this study. Male Parmesan miniature pigs were randomly divided into control, model, Nicardil (1.2 mg/kg), SSNX-Tre(20 mg/kg) and SSNX-Pre(20 mg/kg) groups, with 6 animals in each group. SSNX-Pre group (20 mg/kg) was administered 7 days before MI/RI, whereas the other four groups were fed normally for 7 days.

On the seventh day, all groups except the control group were anesthetized intravenously with 3% sodium pentobarbital (30 mg/kg) at the ear margin, the right common carotid artery was isolated, the distal end was ligated, a 6F arterial sheath tube was placed, and 200 U/kg of heparin was injected from the lateral tube of the arterial sheath, and left coronary angiography was performed with a 6F 35L right coronary guiding catheter placed at the opening of the left coronary artery under C-arm X-ray machine fluoroscopy to observe the distribution of coronary arteries. After the angiogram, an exchange guidewire was placed, and the guidewire was placed in the middle of the anterior descending branch of the coronary artery. Then the balloon was pressurized by 8–12 atm according to the difference of left anterior descending branch(LAD) vessel diameter, and the guidewire and balloon were withdrawn after 30 min to complete the reperfusion [12, 13]. Finally, an electrocardiogram (ECG) was performed at 15 min postoperatively to assess the success of the model construction by ECG changes.

After successful preparation of the model, SSNX (20 mg/kg) was mixed and fed for 7 days in SSNX-Tre and SSNX-Pre groups, Nicardil (1.2 mg/kg) was mixed and fed for 7 days in Nicardil group, and the control and model groups were fed regularly for 7 days.

#### Echocardiography

Echocardiography was performed 7 days after surgery, using a VividS5 colour Doppler ultrasound diagnostic instrument with a high-frequency probe for localisation, and M-mode echocardiography was used to observe cardiac motion in the long-axis and short-axis views of the left ventricle. The left ventricular end-diastolic internal diameter(LVIDd), left ventricular end-systolic internal diameter (LVIDs) were calculated in the long-axis view, and the ejection fraction (EF) and shortening fraction (FS) of cardiac systolic function were calculated.

#### ELISA assay

The blood was collected from the right femoral artery 7 days after surgery. Next, the blood was allowed to stand for 1 h at 4 ℃, centrifuged at 3500 rpm for 10 min. Finally, The supernatant was used to evaluate the content of creatine kinase (CK) lactate dehydrogenase (LDH) and serum cardiac troponin (cTnT) according to the ELISA kit instructions.

#### Measurement of myocardial infarction area

Infarct size was examinate as previously described [14, 15]. Briefy, after reperfusion 30 min, pigs were anesthetized, and the hearts were rapidly excised and rinsed with saline, and the ventricular sections were cut into 3–4 mm thick slices evenly parallel to the coronary sulcus. Next, the slices were immersed in freshly prepared precooled 0.025% nitro-blue tetrazolium (NBT) buffer and incubated at 37 °C for 30 min for NBT staining. The front and reverse sides of the five myocardial slices were scanned with a scanner to obtain the scanned images, and the infarcted area (NBT non-stained area, grayish white) and non-infarcted area (NBT stained area, blue-black) were measured bilaterally in each myocardial slice with a multimedia color pathology image analysis system to calculate the infarcted area (infarcted area/total left ventricular area × 100%).

#### Histopathological change

HE staining and Masson’s trichrome staining were used to detect pathological and morphological changes in the myocardial tissue. One 1 cm^3^ of myocardial tissue was fixed in 10% formalin for 24 h, embedded in paraffin, continuously sectioned (5 μm) on a microtome for HE staining and Masson staining, and then imaged under a light microscope.

### Network pharmacology analysis

#### Collection main chemical components in SSNX and targets prediction

The main chemical components of SSNX were identified by HPLC–MS (Additional file 1: Figure S1, S2 and Tables S1–S2). Next, the 2D structural molecular formulae of the active components were obtained from Pubchem(https://pubchem.ncbi.nlm.nih.gov/) and imported to Swisstargetprediction(http://swisstargetprediction.ch/) for predicting the targets of the main chemical components.

#### Acquisition of intersectional targets for SSNX to treat MI/RI

Targrts associated with MI/RI were collected by casting about the keywords “myocardial ischemia–reperfusion injury” in the databases such as OMIM (https://omim.org/) and GeneCards (https://www.genecards.org/). The score value in Genecards represents the association between the target and the disease; the higher the score value, the closer the target and disease association. In general, targets with a score value larger than the median were considered potential MIRI targets. The potential targets for SSNX in the treatment of MI/RI were obtained by combining the targets of the SSNX and MI/RI.

#### Protein–protein interaction (PPI) network construction

A PPI network was built to elucidate the relationship between the targets of SSNX and MI/RI. The following criteria were used to build the PPI network using String 11.0 (https://string-db.org): organism (Homo sapiens), combined score (> 0.7), and the rest of the settings (default). In order to identify probable protein functional modules and characterize their function by examining the biological processes in which they engage, the PPI network was further examined using the MCODE plugin in Cytoscape 3.9.0.

#### GO enrichment analysis and KEGG pathway analysis

The intersecting genes were uploaded to the Metascape (https://metascape.org/gp/index.html#/main/step1) for GO annotation analysis and KEGG pathway enrichment analysis, to predict the cellular components, molecular functions, biological processes, and pathways of SSNX treatment of MIRI.

#### Molecular docking

Based on the above results, the structures of the pivotal targets were obtained from the PDB database (https://www.rcsb.org) and molecular docking was performed by autodock with the active components of SSNX to observe the binding energy of each group. In general, a lower binding energy indicates a higher degree of compatibility between molecules.

### Effect of SSNX on mitophagy

#### Transmission electron microscopy

The heart tissue was fixed in 3% glutaraldehyde for 2 h. The fixative was aspirated, 1% osmium tetroxide was added, fixed for 1 h, aspirated, stained with uranyl acetate for 1 h, dehydrated in steps of 50–100% alcohol for 15 min each, and pure alcohol: pure acetone (1:1) and pure acetone for 10 min each. Acetone was mixed with epoxy resin at a ratio of 1:1 and permeabilised for 2 h. The epoxy resin was embedded, and the sections were ultra-thinly sectioned at a thickness of 50–70 nm apiece using a Leica ultrathin microscope. The prepared ultra-thin sections were viewed and photographed in a transmission electron microscope after lead staining.

#### Determination of myocardial ATP content

The heart was weighed and added to 9 times the volume of boiling double-distilled water at a ratio of weight/volume = 1:9. A homogenate was made and boiled in boiling water for 10 min, mixed and extracted. The supernatant was centrifuged and assayed using an enzyme marker, and the ATP content of the myocardial tissue in each group was calculated by substituting the formula.

#### Measurement of mitochondrial membrane potential in cardiomyocytes

A portion of the myocardium was excised and weighed, rinsed in saline and then washed twice in 1 ml of Lysis Buffer. After 20 grinding sessions in an ice bath at 0 °C, the tissue was homogenised and divided into centrifuge tubes and centrifuged at 1000 g for 5 min at 4 °C. The supernatant was taken out and placed to a new centrifuge tube and centrifuge again for 5 min. Next, transfer to a new tube and centrifuge at 12,000 g for 10 min at 4 °C. Let the mitochondria settle at the bottom of the tube after removing the supernatant. Add 0.5 mL Wash Buffer to resuspend the precipitate, centrifuge at 1000 g for 5 min at 4 °C and then centrifuge again at 12000 g for 10 min at 4 °C. Discard the supernatant and the precipitate will be high purity mitochondria. The precipitate can be resuspended with 50–100 μL of Store Buffer or reaction buffer and used immediately to detect the mitochondrial membrane potential using the JC-1 fluorescent probe method.

#### Western blot

Myocardial tissue from each group of experimental animals was added to 300 μl of RIPA tissue lysate, 6 μl of protease inhibitor was added to the lysate, left on ice for 1 h, centrifuged at 12000 g for 10 min, and the supernatant was retained. The total protein content was measured using the BCA protein quantification kit. 8% isolate gel and 5% condensation gel were prepared. After the sample was loaded, the electrophoresis was stopped at a constant pressure of 80 V for 30 min, and then at a constant pressure of 120 V until the bromophenol blue ran below the platinum metal wire in the electrophoresis tank. After washing the membrane, incubated with primary and secondary antibodies respectively. The ECL chemiluminescence method was used and the relative grey values of the proteins were calculated using Image J software.

### Statistical analysis

All data presentations are shown as mean ± standard deviation (SD). Oneway analysis of variance (ANOVA) was carried out for comparing multiple groups when the data were compounded with a normal distribution using SPSS 23.0 (IBM, USA). Diferences were considered significant at P < 0.05. Origin 2021 (GraphPad Software, USA) was utilized for graphing.

## Result

### Experimental protocols and evaluation of miniature pigs MI/RI models

**Fig. 1:**
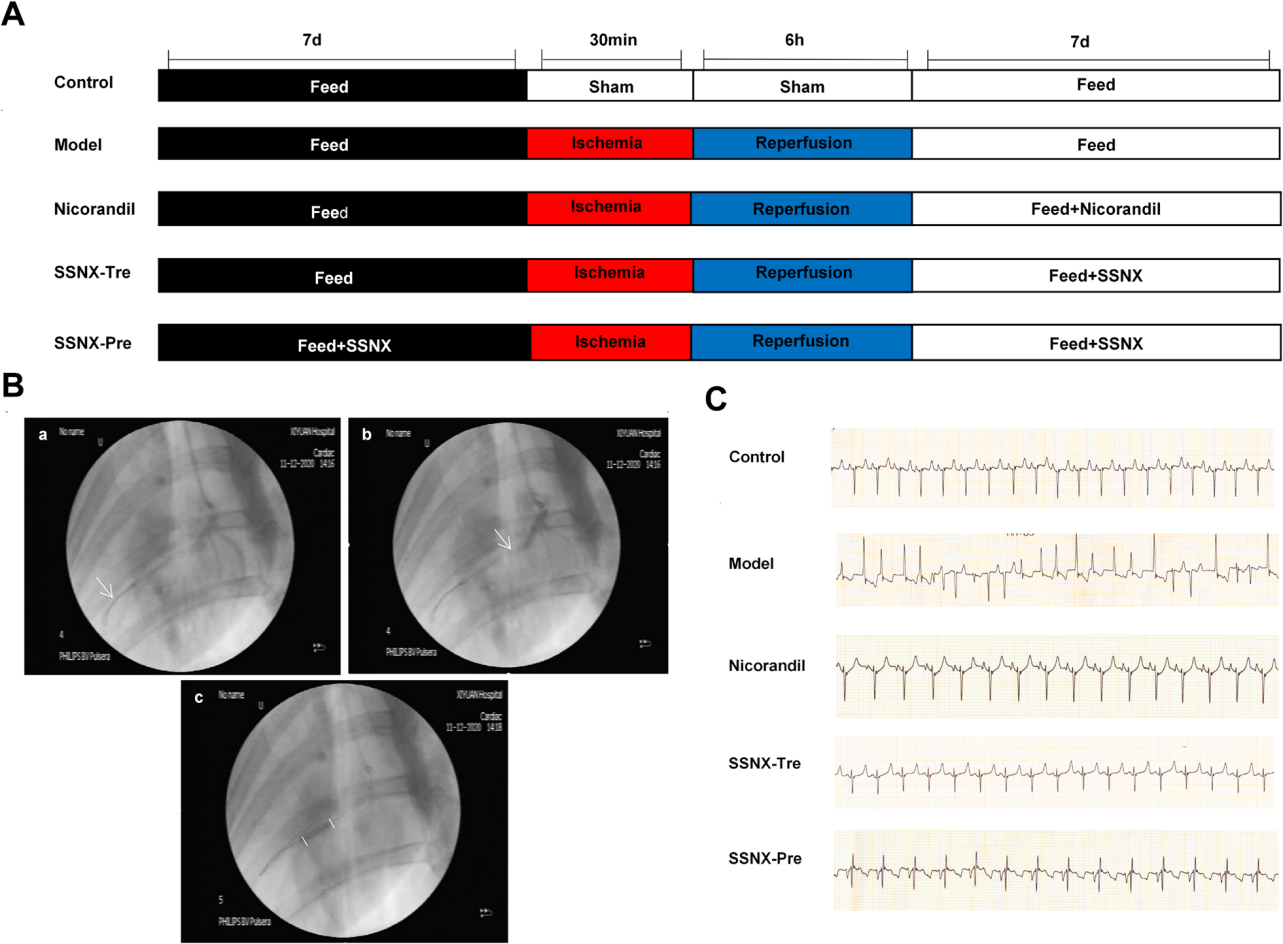
Experimental flowchart and the establishment of MI/RI minpig model. **A** Timeline of model building and drug delivery.** B** The photographs of coronary angiography in different periods. **a** Coronary angiography was performed and white arrow indicates the location of the guidewire. **b** Placement of guidewires in LAD and white arrow indicates the narrowest part of the coronary artery. **c** Balloon dilation LAD and segment between the two white lines is the stent implantation section. **C** The representative photographs of electrocardiograms after MI/RI

A total of 30 male miniature pigs were included in this experiment and randomly divided into control group, model group, Nicorandil group, SSNX-Tre group and SSNX-Pre group, with 6 animals in each group (Fig. 1A). Coronary angiography was performed during the construction of the model to observe the coronary artery LAD embolism in miniature pigs (Fig. 1B). ECG changes were also observed at each stage during the construction of the model. This was used as a criterion to determine the success of the MI/RI model: during the MI/RI process, pathological ECG changes such as ST-segment elevation, pathological Q-wave or T-wave inversion and ground level were all seen on the lead II ECG to varying degrees, which was used to determine successful modelling (Fig. 1C).

### SSNX significantly attenuates MIRI in miniature pigs

**Fig. 2:**
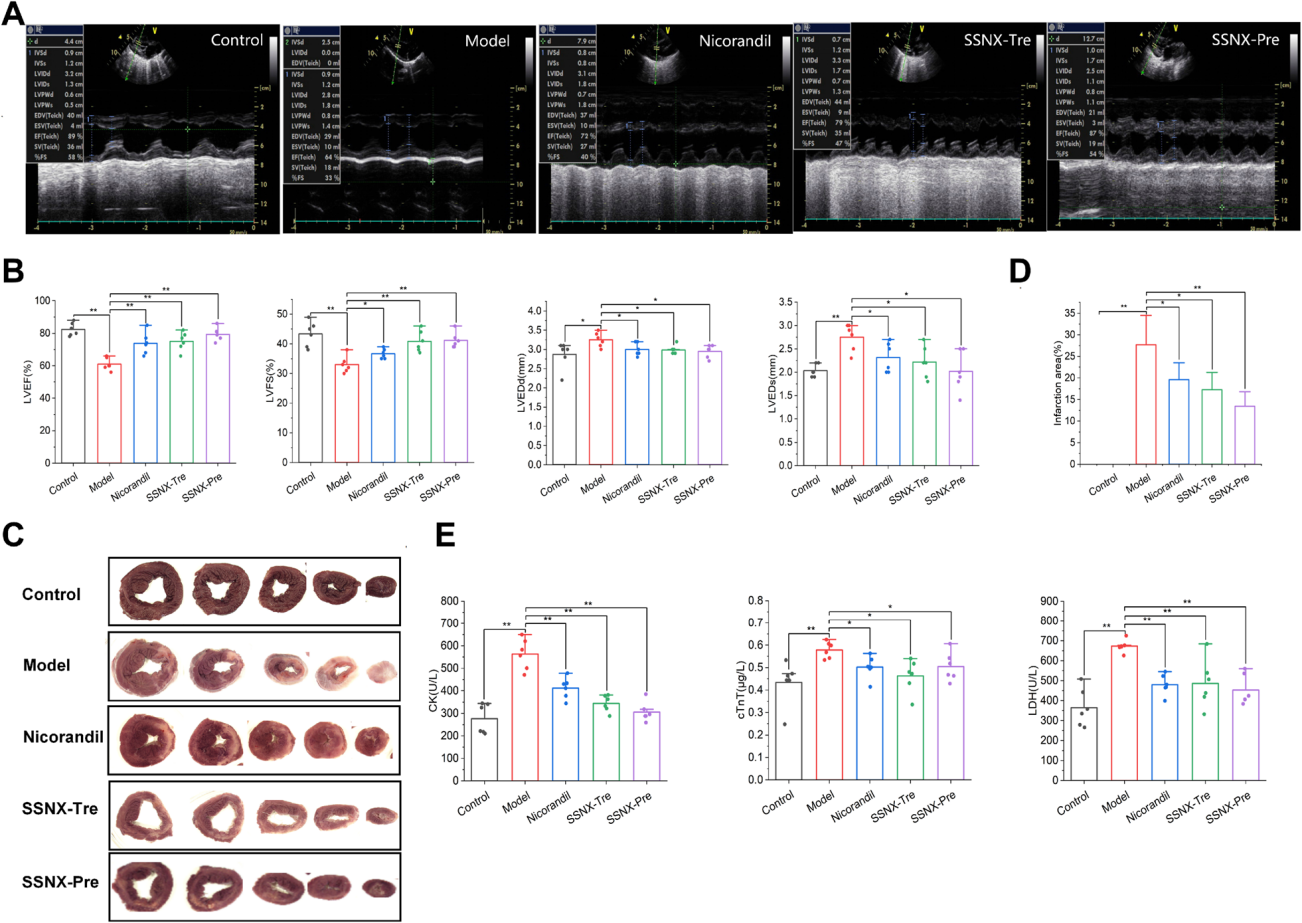
SSNX significantly attenuates MI/RI in miniature pigs. **A** Representative M-mode echocardiography tracings. **B** Quantification parameters of cardiac function assessment including ejection fraction (LVEF), fractional shortening (LVFS),left ventricular end diastolic dimension (LVEDd) and left ventricular end-systolic dimension (LVEDs) (n = 6). **C**, **D** NBT staining and histogram of infarct size of myocardial tissue (n = 5). **E** The cTnT content and activities of CK and LDH (n = 6). *P < 0.05, **P < 0.01

In order to test the cardioprotective effect of SSNX, cardiac function was assessed via echocardiography at 7 days following MIRI. The results indicated that LVEDs were significantly higher, EF and FS were significantly lower in the model group compared to the control group; LVEDd and LVEDs were significantly decreased and EF and FS were significantly increased in the Nicorandil group, SSNX-Tre group and SSNX-Pre group compared to the model group. In addition, all cardiac function indexes the SSNX-Pre group were better than those in the SSNX-Tre group and Nicorandil group, and all cardiac function indexes the SSNX-Tre group were slightly better than Nicorandil group, suggesting that SSNX can reduce the impairment of cardiac function in MIRI miniature pigs to some extent (Fig. 2A–B).

The NBT staining results showed that the increase in myocardial infarct area was particularly significant in the model group compared with the control group; compared with the model group, the myocardial infarct area in the Nicorandil group and SSNX-Tre group showed different degrees of reduction; the myocardial infarct area in the SSNX-Pre group was more obvious than that in the Nicorandil group and SSNX-Tre group (Fig. 2C–D), suggesting that SSNX has a certain function in improving the infarct status of MI/RI miniature pigs.

In addition, compared with the control group, the serum CK, cTnT and LDH contents in the model group increased significantly; compared with the model group, the serum CK, cTnT and LDH contents in the Nicorandil group, SSNX-Tre group and SSNX-Pre group all decreased to different degrees (Fig. 2E), which proved that SSNX could reduce the extent of myocardial injury in miniature pigs.

### SSNX improves myocardial tissue pathological structure, and reduces myocardial fibrosis

**Fig. 3:**
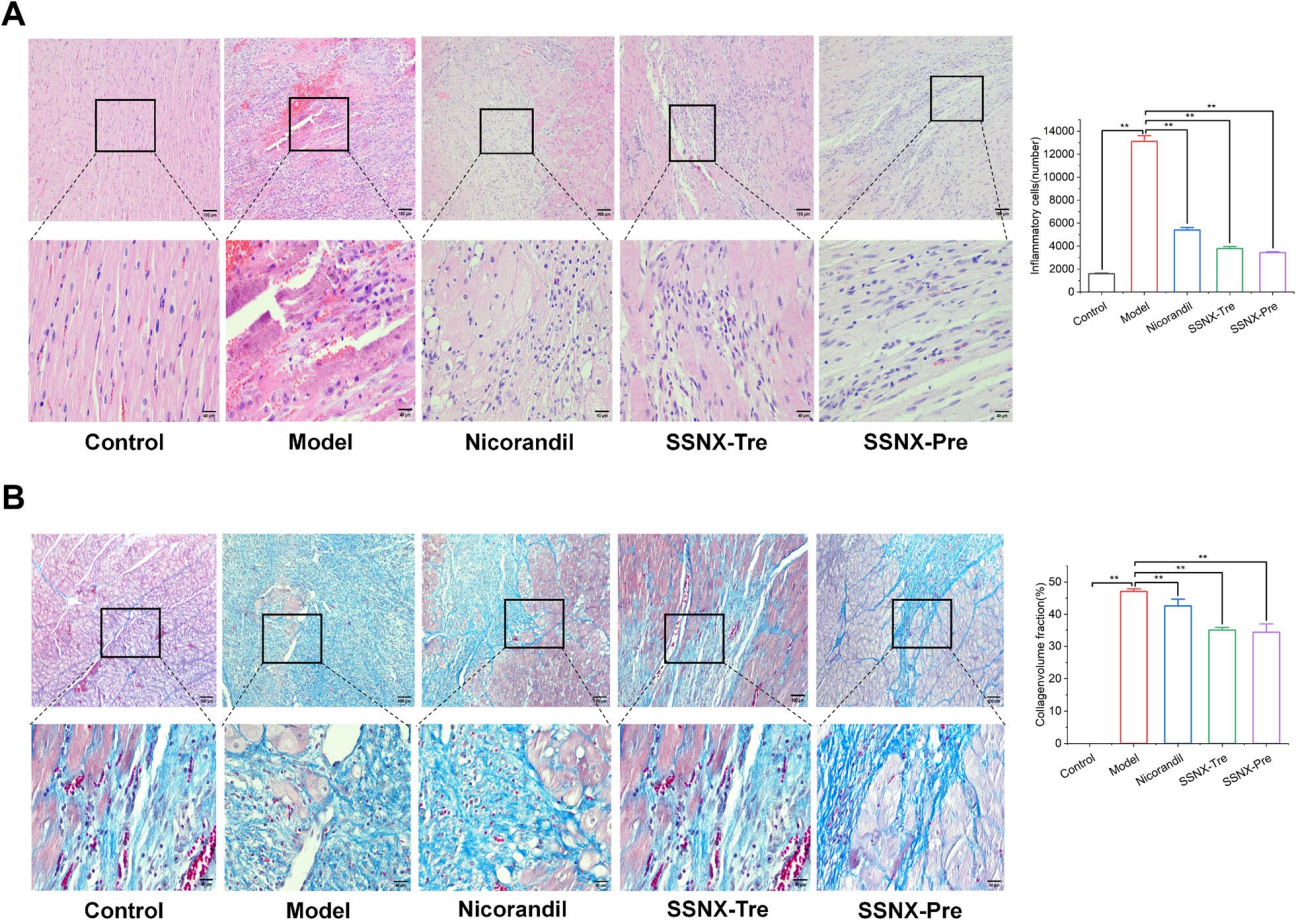
SSNX attenuates myocardial necrosis and fibrosis in MI/RI minpigs. **A** HE staining and semi-quantitative analysis of myocardial tissue sections. Scale bar = 100 μm, Scale bar = 40 μm (n = 3). **B** Masson trichrome staining and semi-quantitative analysis of myocardial tissue sections. Scale bar = 100 μm, Scale bar = 40 μm (n = 3)

The paraformaldehyde-fixed heart sections were stained with HE and Masson staining to observe the myocardial histopathology. In the control group, myocardial fibres were neatly aligned with each other, the nuclei of myocardial cells were round and centrally located, and there was no degenerative necrosis or swelling of myocardial fibres, and no inflammatory cell infiltration; the model group showed severe inflammatory cell infiltration, disturbed arrangement of myocardial cells, and increased fibrosis in the surrounding tissues of the infarcted area; in the SSNX-Tre group, patches of myocardial fibres were degenerative necrosis in some myocardial fibres, accompanied by partial fibrous tissue hyperplasia and inflammatory. In the SSNX-Pre group, most of the myocardial fibres were aligned with each other, and some of the myocardial fibres were degenerated and necrotic, with a small amount of fibrous tissue hyperplasia and inflammatory cell infiltration (Fig. 3).

### Collection of potential targets and construction of PPI networks

**Fig. 4:**
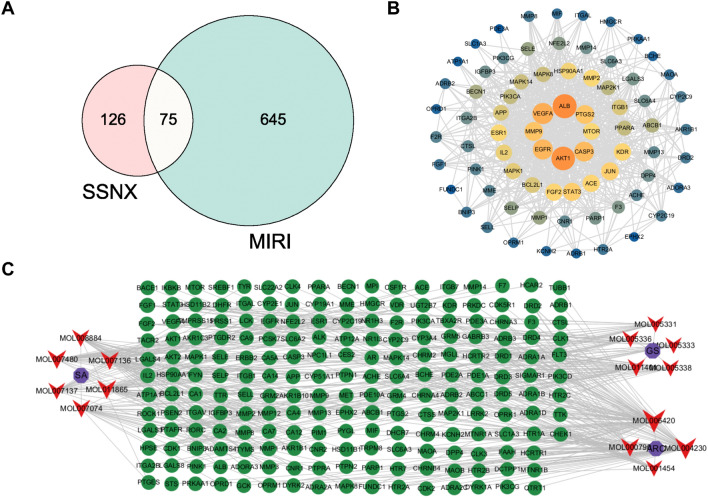
Network analysis of SSNX treating MI/RI. **A** Venn diagram of 75 common targets of SSNX and MI/RI. **B** PPI network diagram of common targets. **C** Diagram of SSNX-Compounts-Target network. Purple nodes refer to the total ginsenosides (GS), total salvianolic acids (SA) and total alkaloids of rhizoma corydalis (ARC); Red nodes refer to the blood compounds; Green nodes refer to the targets

The Swisstargetprediction database was used to match the targets of 27 compounds discovered by HPLC–MS, with a total of 15 active components and 201 targets matched. MI/RI targets were obtained from the GeneCards database in descending order of relevance score, and the median was utilized to obtain 645 MI/RI targets. The drug targets and disease targets were then imported into the Venny 2.1.0 (https://bioinfogp.cnb.csic.es/tools/venny/) and analysed to obtain a total of 75 intersecting genes, which are the potential targets for SSNX to treat MI/RI (Fig. 4A).

In order to evaluate the characteristic of common targets, 75 intersecting genes were imported into the STRING database to produce PPI network. The PPI network results were imported into Cytoscape 3.9.0 software to build a PPI network map (Fig. 4B). A drug-component-target network diagram was also constructed (Fig. 4C), this suggests that SSNX treats MI/RI through multi-targeting.

### GO and KEGG enrichment analysis

**Fig. 5:**
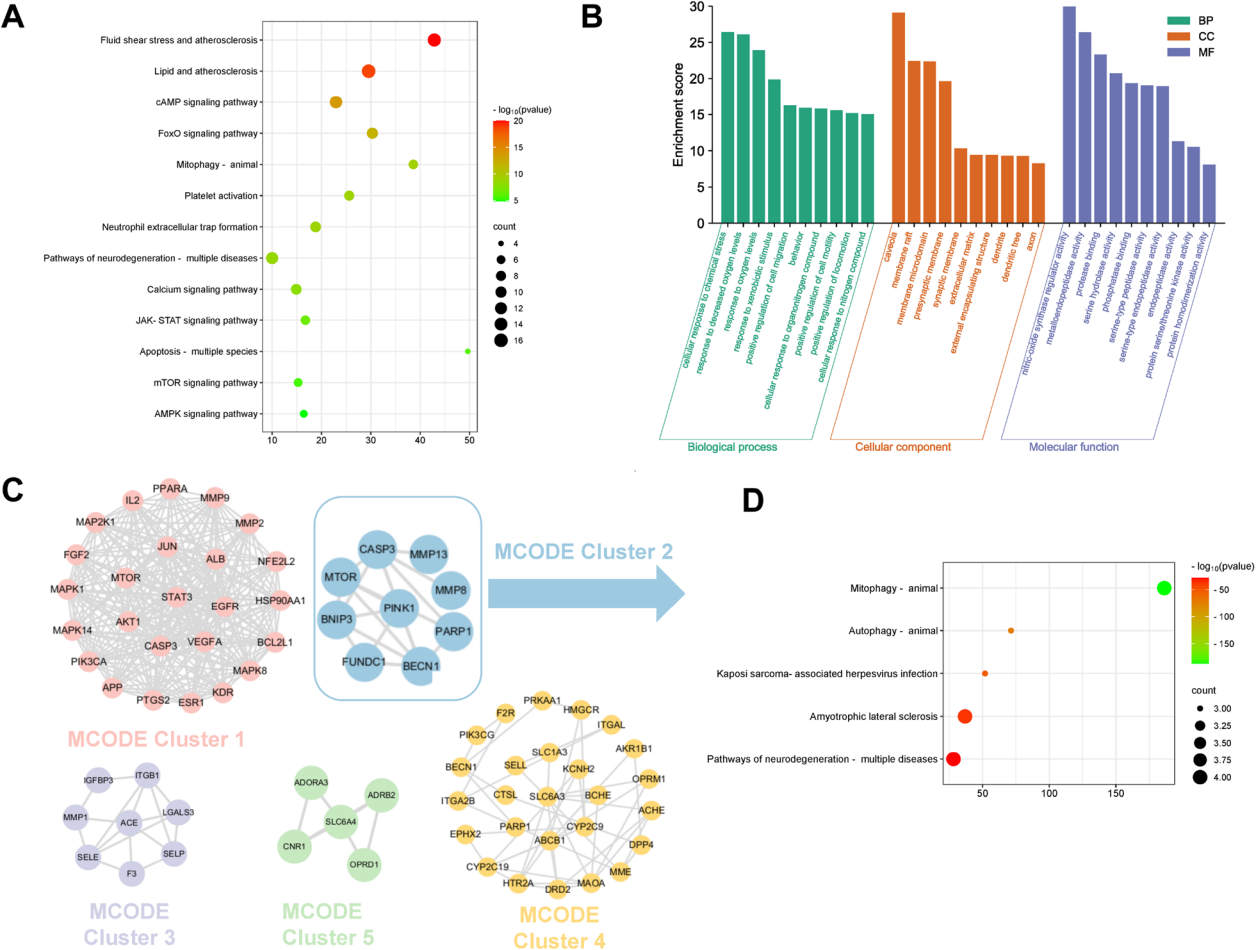
Enrichment and modular analysis of the common targets. **A** KEGG analysis of SSNX anti-MI/RI targets. **B** GO analysis of SSNX anti-MI/RI targets. **C** Derived different MCODE clusters. D The enriched terms of MCODE cluster 2 in KEGG pathways

GO function and KEGG pathway enrichment analysis were performed through the Metascape to clarify the potential functions of 75 overlapping targets. KEGG analysis suggested that SSNX was highly linked with fluid shear stress and atherosclerosis, lipid and atherosclerosis, cAMP signaling pathway, FoxO signaling pathway and mitophagy—animal (Fig. 5A). According to the GO analysis, the caveola, membrane raft and membrane microdomain are the main targets of SSNX's impacts on cell components (Fig. 5B). In addition, SSNX played a role in a variety of biological processes, mainly including cellular response to chemical stress, response to decreased oxygen levels, response to oxygen levels and so on (Fig. 5B). Furthermore, SSNX was mainly associated with nitric-oxide synthase regulator activity, metalloendopeptidase activity, protease binding and other molecular functions (Fig. 5B).

To further investigate the underlying mechanisms, the MCODE algorithm was utilized to find highly relevant network targets from the PPI network, yielding five significant clusters (Fig. 5C). Among them, MCODE Cluster 2 enriched on mitophagy—animal (Fig. 5D) and contained key targets that regulate mitophagy: PINK1, Parkin, FUNDC1 and BNIP3. Therefore, the mitophagy pathway was selected for the next step of experimental validation in this study.

### Molecular docking

**Fig. 6:**
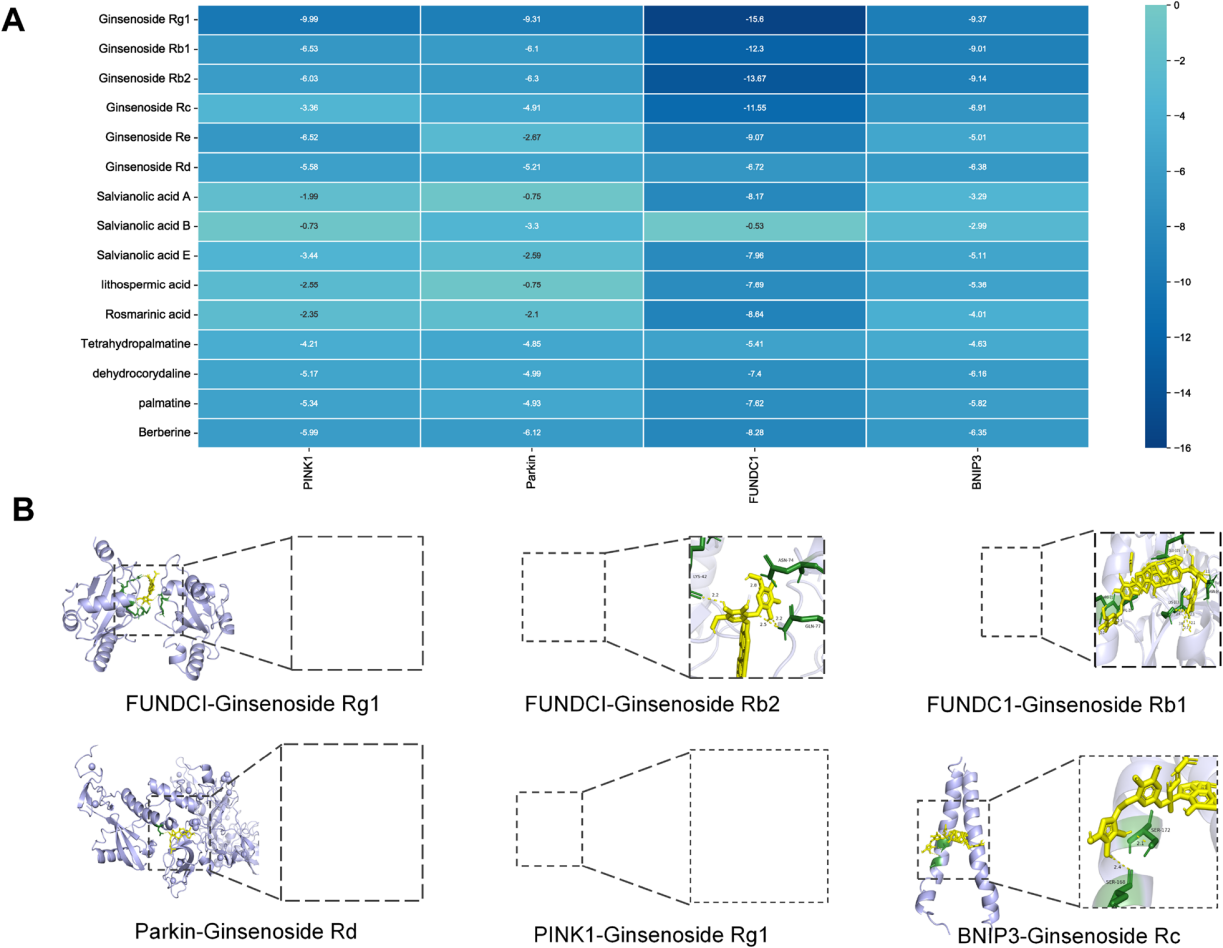
Molecular docking results. **A** The heatmap of docking scores of mitophagy-related targets combining to 15 blood compounds of SSNX. **B** The representative docking complex of key targets and compounds

Based on the results of enrichment analysis and MCODE algorithm analysis, the mitophagy-related targets PINK1, Parkin, FUNDC1 and BNIP3 were selected to dock with the active components of SSNX (Fig. 6A). Generally, the molecular docking results were less than − 5.0 kcal/mol, indicating well binding activity [16]. The lower the binding energy, the stronger the binding ability of the molecules. The results showed that 63% of the docking combinations had good binding activity, with FUNDC1 having the best docking results.

### SSNX can improve mitochondrial function and structure in MI/RI miniature pigs

**Fig. 7:**
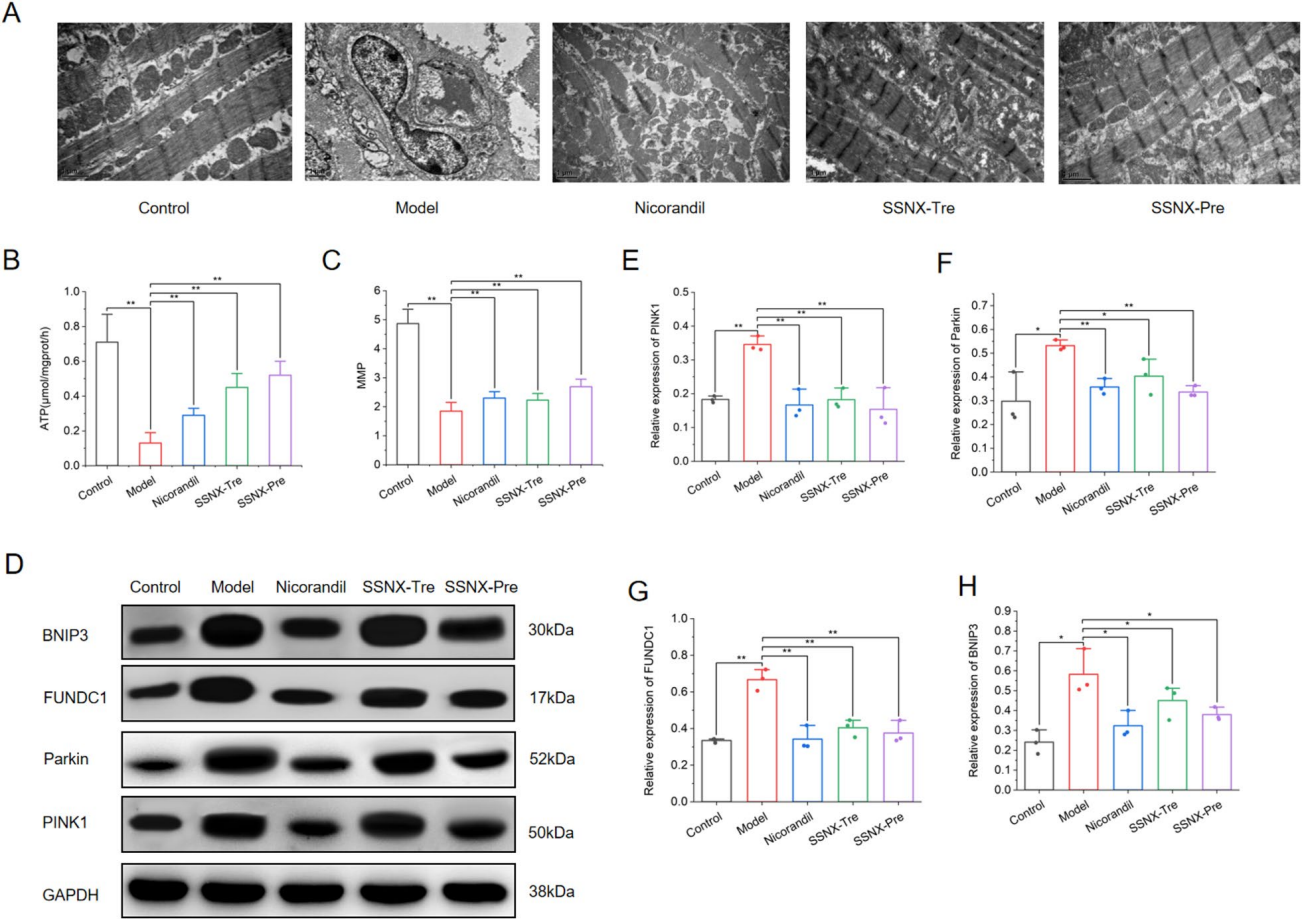
SSNX improves mitochondrial function and inhibits mitophagy. **A** Representative TEM image of mitochondrial ultrastructure. Scale bar = 1 μm. **B** ATP content in myocardial tissue of minipigs in each group (n = 6). **C** Changes in mitochondrial membrane potential in myocardial tissue of minipigs in each groups (n = 6). **D**, **E** Representative western blot images and bar graphs of the relative expressions of PINK1,Parkin,FUNDC1 and BNIP3 in each group (n = 3). *P < 0.05, **P < 0.01

According to the result of network pharmacology, we further investigated whether SSNX attenuates MI/RI through the mitochondrial pathway. Transmission electron microscopy shows that the myocardial filaments were obviously broken or loosened, the mitochondria were swollen and enlarged or vacuolated, the internal structure was loose, and autophagosomes were formed in the myocardial tissue and mitochondria in the model group; myocardial myofilaments in the Nicorandil group, SSNX-Tre group and SSNX-Pre group were broken or loosened but to a significantly lesser extent than in the model group, mitochondria were swollen and enlarged but relatively intact, cristae were less loosened, there was no significant overall vacuolization, and there were a few autophagosomal structures in myocardial tissue and mitochondria(Fig. 7A).

In addition, compared with the control group, the myocardial ATP content and MMP in the model group were significantly reduced, which were statistically different; compared with the model group, the myocardial ATP content and mitochondrial membrane potential in the SSNX groups were significantly increased, although there was a trend of increase in Nicorandil group, but the increase was not as obvious as that in the SSNX groups (Fig. 7B–C). The above results indicated that SSNX can improve mitochondrial function and structure in MI/RI miniature pigs.

### SSNX inhibits mitophagy in MI/RI miniature pigs

According to the result of molecular docking, the active ingredient of SSNX has good binding activity to mitophagy-related proteins. Therefore, western blot was further to determine the protein expression of targets associated to mitophagy (PINK1, Parkin, BNIP3, and FUNDC1). In comparison to the control group, the proteins expression of PINK1, Parkin, BNIP3 and FUNDC1 were significantly higher in the model group. However, the relative expression of the above proteins was significantly lower in the Nicorandil and SSNX-Pre groups compared to the model group, and FUNDC1 and Parkin were significantly lower in the SSNX-Tre group, while PINK1 and BNIP3 were not significantly different. Taken together, SSNX exerted a protective effect on the function and structure of MI/RI miniature pigs by inhibiting mitophagy (Fig. 7D–H).

## Discussion

MI/RI is a pathological process that occurs when blood flow is restored to the ischaemic myocardial tissue while causing stress injury. Myocardial I/R injury is associated with severe clinical manifestations leading to a high incidence of cardiac insufficiency and heart failure [17]. There are no effective methods to reduce MI/RI, so early intervention is particularly important, and TCM has unique advantages in this regard [18]. However, due to the multi-component and multi-target nature of Chinese medicine, the mechanism of traditional pharmacological methods in the treatment of MI/RI is still difficult to be fully elucidated. Therefore, in this study, we first validated the efficacy of SSNX through animal experiments, and then investigated the potential mechanism of SSNX intervention in MIRI through network pharmacology. In addition, molecular docking methods and further target validation demonstrated that the protective effects of SSNX on MIRI in miniature pigs, possibly through the inhibition of mitophagy.

Previous studies found that Nicorandil, a nitrite-like antianginal drug used as an ATP-sensitive potassium channel (K^+^_ATP_) opener and a potent activator of guanylyl cyclase, mediates protection against MI/RI [19]. Nicorandil has a preconditioning-like cardioprotective effect against ischemic injury in myocardial infarction patients [20]. Futhermore, Nicorandil reduced the area of myocardial infarction, improved cardiac function, and protected the damaged myocardium after ischemia–reperfusion. In our study, the results demonstrated that Nicorandil and SSNX significantly improved cardiac function and attenuated myocardial I/R injury, improved myocardial histopathological structure and reduced myocardial fibrosis in MI/RI miniature pigs. To further explore the potential mechanism, we obtained 75 potential targets of SSNX for the treatment of MI/RI by network pharmacology. Additionally, the KEGG results offer a relatively complete and macroscopic mechanistic picture of SSNX for the treatment of MI/RI. To further explore more specific mechanisms, we analysed modularly by the MCODE algorithm. The results of the analysis grouped the potential targets into five clusters representing different functions, and we selected cluster 2 for subsequent studies. Enrichment analysis of cluster 2 suggested that the key mechanism of SSNX for MI/RI may be related to mitophagy. Interestingly, the molecular docking results also further confirm this idea.

According to the molecular docking results, ginsenoside Rg1, Rb1 and Rb3 displayed good binding to mitophagy-related proteins, with the best binding to FUNDC1. Subsequently, the literature was queried to see if these three key compounds could treat MI/RI by modulating mitophagy. Research showed that Ginsenoside Rg1 significantly improved cardiac remodelling in left anterior descending coronary artery ligated mice, as evidenced by a reduction in cardiac fibrosis accompanied by an improvement in cardiac function. The mechanism was that ginsenoside Rg1 significantly increased mitochondrial formation, improved cardiac mitochondrial damage and enhanced SIRT1/PINK1/Parkin-mediated mitophagy during cardiac remodeling [21] However, the effects of the other two active components on mitophagy have not been investigated, and therefore further experimental validation of the above monomers is required to reveal their effects and mechanisms.

Based on the above predicted results, we further understood the effect of SSNX on the function of mitochondria. Transmission electron microscopy observed that SSNX significantly improved mitochondrial morphology. Interestingly, SSNX not only protected mitochondrial morphology, but also protected mitochondrial function by increasing myocardial ATP content and maintaining mitochondrial membrane potential. It is suggested that SSNX may protect mitochondrial function and morphology, which in turn interferes with MI/RI. Based on these results, we further verified whether SSNX could affect mitophagy.

Autophagy is a metabolic process that degrades macromolecules and can degrade damaged organelles within cells, specific macromolecules, as well as invading viruses and bacteria [22]. Mitophagy is a type of autophagy that occurs in the mitochondria and is a key process for detecting mitochondrial function and removing damaged or unwanted mitochondria [23]. On the one hand, moderate mitophagy removes damaged or aged mitochondria from the cell, which helps to maintain intracellular homeostasis, achieve mitochondrial renewal and prevent further myocardial damage; on the other hand, over-activated mitophagy increases cell death and aggravates I/R damage [24]. In our experiments, mitophagy was excessively enhanced in the MI/RI miniature pigs model, as evidenced by abnormal mitochondrial structure, reduced ATP production, impaired MMP and a significant increase in mitophagy-related proteins.

Mitophagy is mainly divided into ubiquitin-dependent and non-ubiquitin-dependent receptor pathways, of which PINK1/Parkin is the most typical and is a key pathway regulating mitophagy [25]. Under normal conditions, PINK1 crosses the mitochondrial membrane to enter the mitochondria for degradation, but when the mitochondria are damaged, PINK1 transit into the mitochondria is blocked and accumulates in the outer mitochondrial membrane, which in turn activates Parkin, phosphorylates Parkin and promotes the activation of Parkin E3 ubiquitinylated ligase, which completes the activation of Parkin to recruit junction proteins for the mitochondria that need to be degraded, after which the junction protein LIR is activated with the help of the activated Parkin then binds to LC3 and mediates mitophagy through the LIR structure of the junction protein [26]. A variety of receptor proteins, in addition to PINK1/Parkin, are located in the outer mitochondrial membrane and interact with LC3 through the LIR motif to start mitochondrial autophagy. These proteins include FUNDC1, BNIP3, and NIX. FUNDC1 is an important receptor protein located in the outer mitochondrial membrane, and when mitochondria are damaged, the LIR motif in FUNDC1 activates mitophagy by molecularly docking with LC3 protein through hydrophobic interaction [27]. BNIP3 is located in the outer mitochondrial membrane and contains a transmembrane domain with the C-terminus inserted into the outer mitochondrial membrane and the N-terminal storm in the cytoplasm. BNIP3 relies on the direct action of the transmembrane domain LC3 to mediate the onset of mitophagy [28]. In the MI/RI miniature pig model, SSNX treatment resulted in a significant reduction in FUNDC1 and BNIP3. These results suggest that SSNX can prevent MIRI by inhibiting the PINK1/Parkin pathway and FUNDC1 and BNIP3 receptor-mediated mitophagy.

In summary, network pharmacology and animal experiments demonstrated that SSNX can effectively reduce myocardial I/R injury and protect mitochondrial structure and function, and its effects may be achieved by inhibiting mitophagy. Furthermore, according to the molecular docking results, ginsenoside Rg1, ginsenoside Rb1 and ginsenoside Rb2 are key components associated with mitophagy. The results of this study provide preclinical evidence for SSNX in the treatment of MI/RI and provide a more novel perspective for the treatment of MI/RI with Chinese herbal medicine.

### Supplementary Information

**Additional file 1: Fig. S1.**Positive ion mass spectrometry of SSNX.** B** Negative ion Mass spectrometryof SSNX. (1) Tetrahydrocolumbamine (2) Tetrahydrojatrorrhizine (3) Protopine (4) Allocryptopine (5) Glaucine (6) Tetrahydropalmatine (7) Columbamine (8) Jatrorrhizine (9) Canadine (10) Corydaline (11) Worenine (12) Berbine (13) Palmatine (14) SAB (15) Dehydrocorydaline. **Fig S2.**Positive ion mass spectrometry of the standard. **B** Negative ion Mass Spectrometry of Standards. (1) catecholamine (2) RA (3) SAB (4) SAA (5) Rd (6) Rf (7) Rg1 (8) Rb1 (9) Rc (10) Rb3 (11) Rb2 (12) Re.

## Data Availability

All data in this study are available from the corresponding author.

## Abbreviations

ATP: Adenosine triphosphate
BNIP3: Bcl-2/E1B-19 kDa interacting protein 3
CHD: Coronary heart disease
CK: Serum creatine kinase
CK-MB: Creatine kinase-MB
cTnT: Serum cardiac troponin
ECG: Electrocardiogram
FUNDC1: FUN14 domain containing 1
GO: Gene ontology
HE: Hematoxylin eosin
I/R: Ischemia/reperfusion
KEGG: Kyoto Encyclopedia of genes and genomes
LDH: Lactate dehydrogenase
LAD: Left anterior descending branch
MI/RI: Myocardial ischemia/reperfusion injury
NIX: NIP3-like protein X
NBT: Nitro-blue tetrazolium
OMIM: Online Mendelian inheritance in man
PPI: Protein–protein interaction
PINK1: PTEN-induced putative kinase 1
SSNX: Shuangshen Ningxin capsule
