# Supplementary material for: Shuangshen Ningxin capsule alleviates myocardial ischemia–reperfusion injury in miniature pigs by modulating mitophagy: network pharmacology and experiments in vivo
Source: Chin Med. 2023 Sep 20;18:120. doi: 10.1186/s13020-023-00810-z (PMC10510173; doi:10.1186/s13020-023-00810-z)

**Fig.1S A** Positive ion mass spectrometry of SSNX. **B** Negative ion Mass spectrometryof SSNX.
1.Tetrahydrocolumbamine 2.Tetrahydrojatrorrhizine 3.Protopine 4.Allocryptopine 5.Glaucine 6.Tetrahydropalmatine 7.Columbamine 8.Jatrorrhizine 9.Canadine 10.Corydaline 11.Worenine 12.Berbine 13.Palmatine 14.SAB 15.Dehydrocorydaline


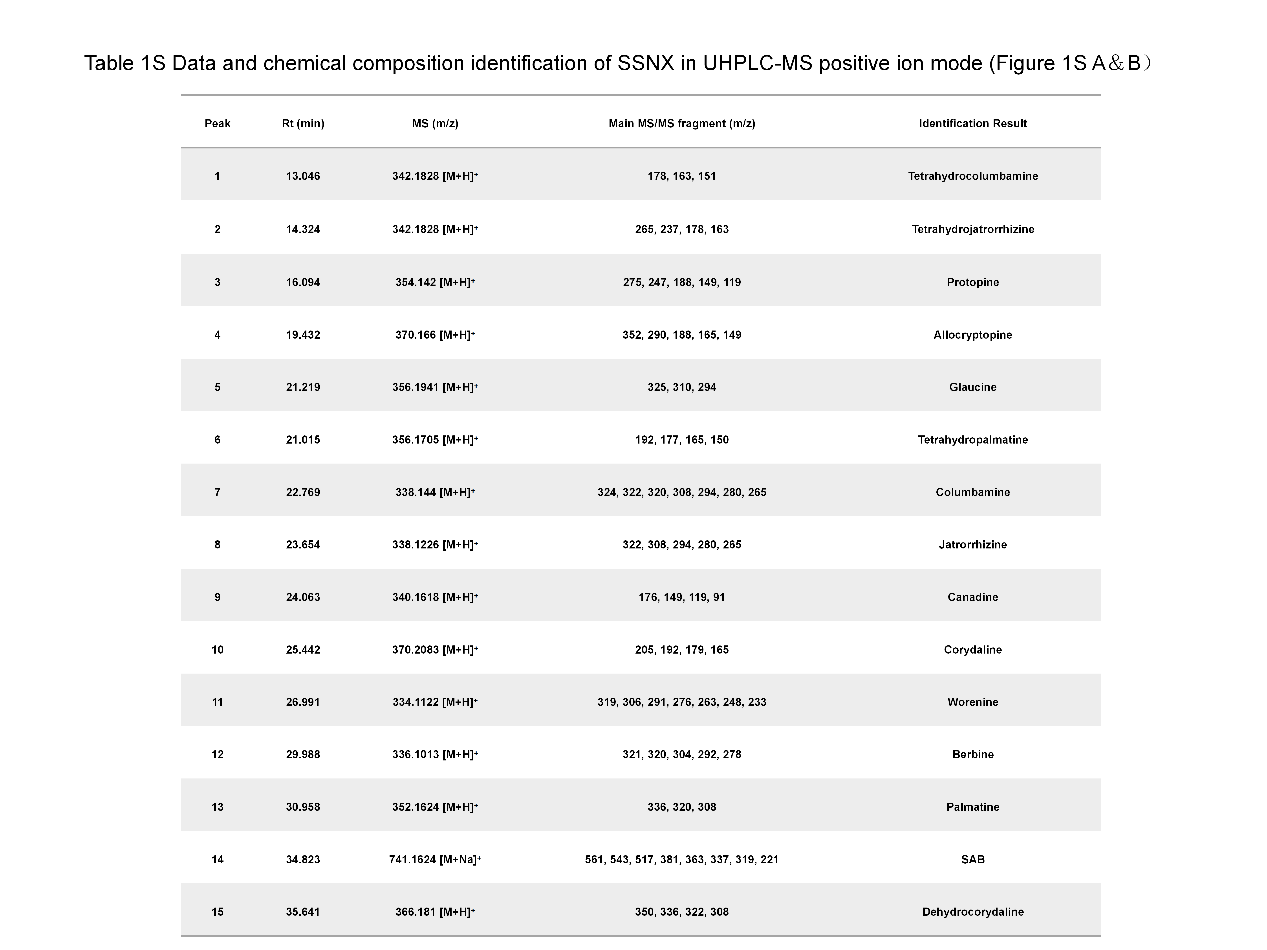


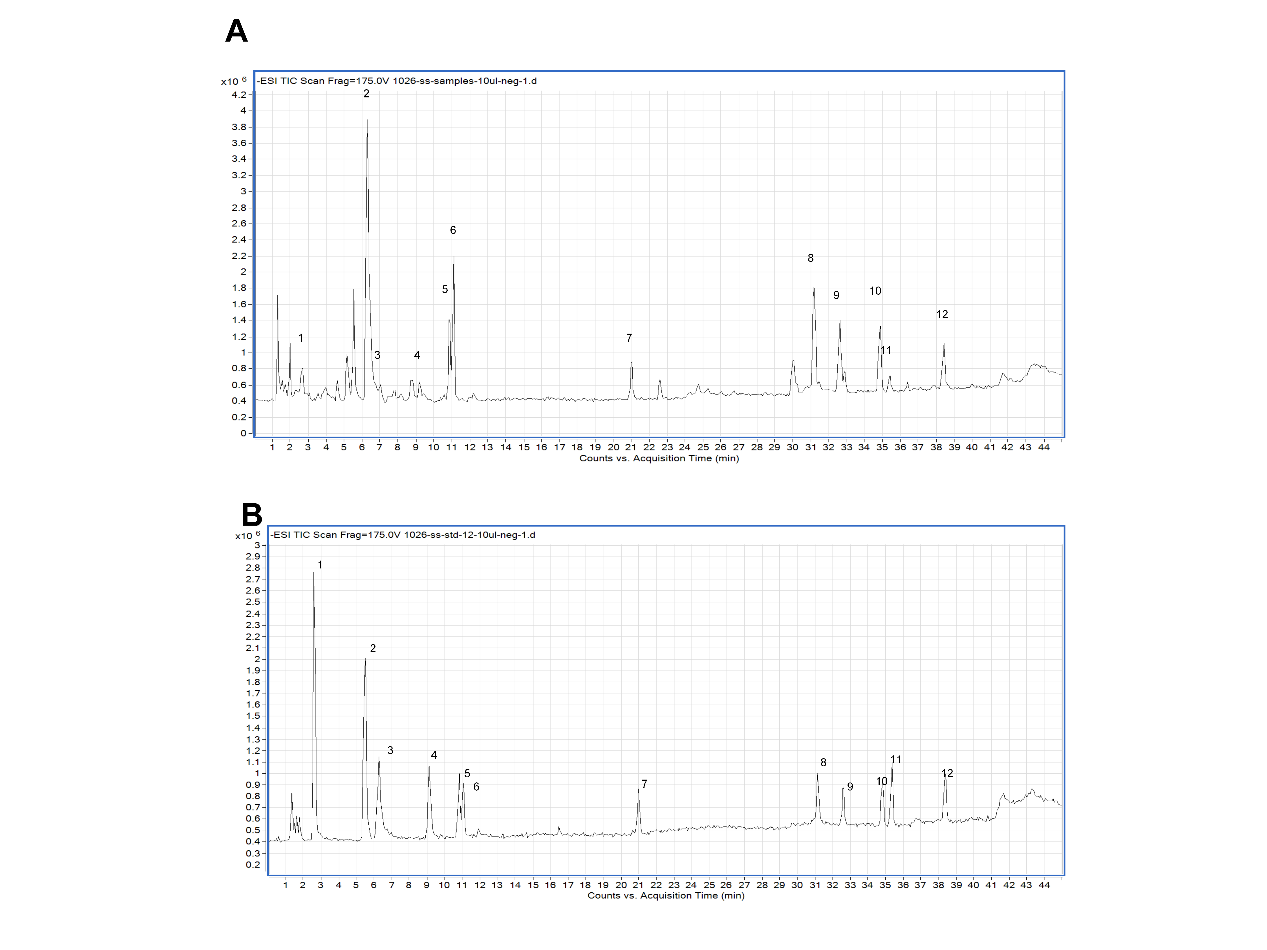


**Fig.2S A** Positive ion mass spectrometry of the standard. **B** Negative ion Mass Spectrometry of Standards. 1.catecholamine 2.RA 3.SAB 4.SAA 5.Rd 6.Rf 7.Rg1 8.Rb1 9.Rc 10.Rb3 11.Rb2 12.Re

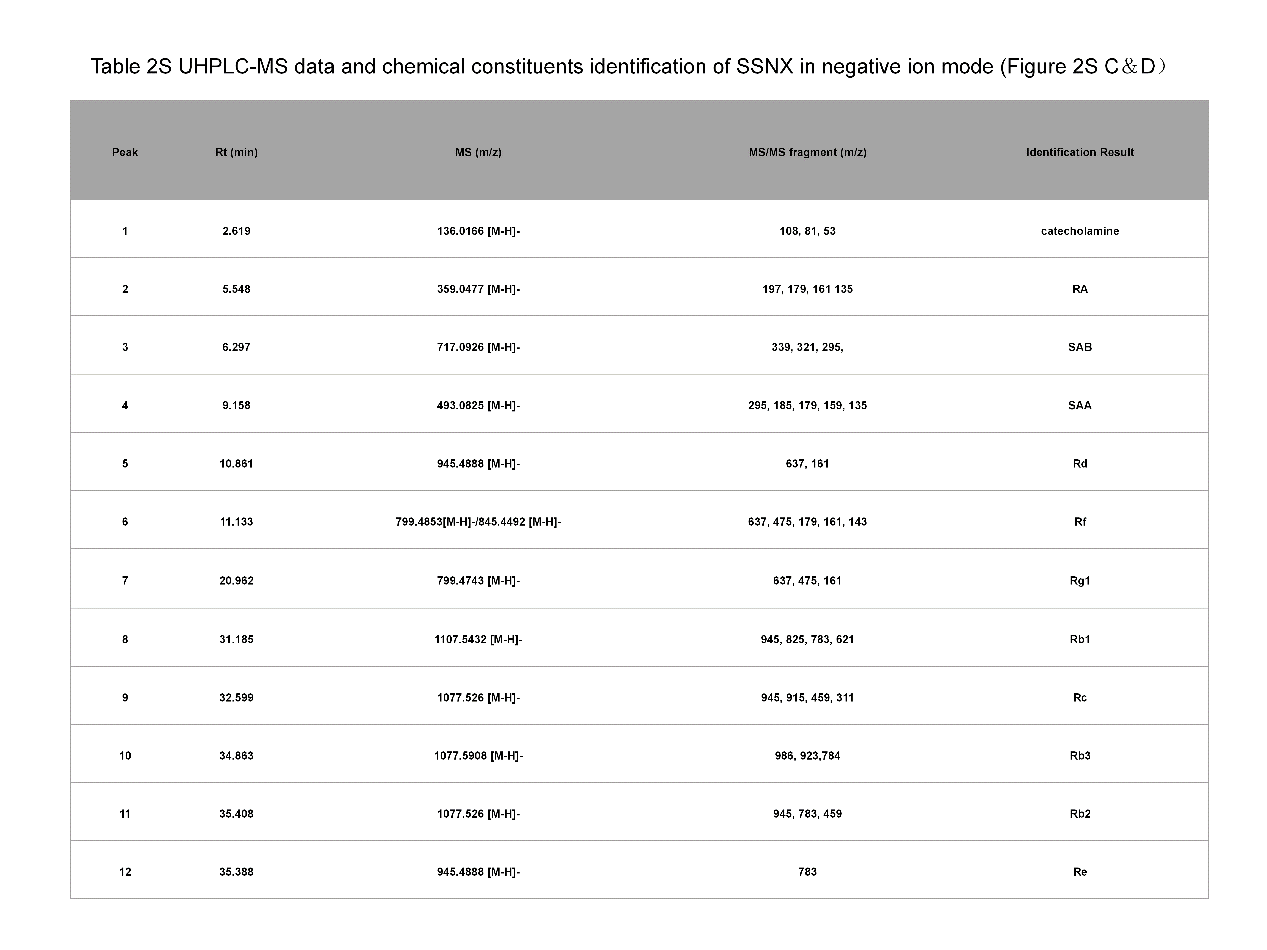

Supplement: Supplementary file 1 — Additional file 1: Fig. S1.Positive ion mass spectrometry of SSNX. B Negative ion Mass spectrometryof SSNX. (1) Tetrahydrocolumbamine (2) Tetrahydrojatrorrhizine (3) Protopine (4) Allocryptopine (5) Glaucine (6) Tetrahydropalmatine (7) Columbamine (8) Jatrorrhizine (9) Canadine (10) Corydaline (11) Worenine (12) Berbine (13) Palmatine (14) SAB (15) Dehydrocorydaline. Fig S2.Positive ion mass spectrometry of the standard. B Negative ion Mass Spectrometry of Standards. (1) catecholamine (2) RA (3) SAB (4) SAA (5) Rd (6) Rf (7) Rg1 (8) Rb1 (9) Rc (10) Rb3 (11) Rb2 (12) Re. [file 13020_2023_810_MOESM1_ESM.docx]
